# SMART Family and Friends Mutual‐Help Groups: Findings From a Mixed‐Methods Formative Implementation Evaluation

**DOI:** 10.1111/dar.70137

**Published:** 2026-03-09

**Authors:** Alison K. Beck, Rebecca M. Gray, Megan Wells, Frank P. Deane, Briony Larance, Leanne Hides, Victoria Manning, Amanda L. Baker, Anthony Shakeshaft, Elizabeth Dale, Angela Argent, Peter J. Kelly

**Affiliations:** ^1^ School of Psychology, Faculty of Arts, Social Sciences and Humanities University of Wollongong Wollongong Australia; ^2^ Tasmanian Centre for Mental Health Service Innovation, Tasmanian Health Service Hobart Australia; ^3^ School of Psychological Sciences, University of Tasmania Hobart Australia; ^4^ National Centre for Youth Substance Use Research, School of Psychology, The University of Queensland Brisbane Australia; ^5^ Eastern Health Clinical School, Faculty of Medicine, Nursing and Health Sciences Monash University Melbourne Australia; ^6^ Turning Point, Eastern Health Melbourne Australia; ^7^ National Drug and Alcohol Research Centre, UNSW Sydney Sydney Australia; ^8^ UQ Poche Centre for Indigenous Health, Faculty of Health and Behavioural Sciences The University of Queensland Brisbane Australia; ^9^ School of Medical, Indigenous and Health Sciences, Faculty of Science, Medicine and Health University of Wollongong Wollongong Australia; ^10^ Formerly of SMART Recovery Australia Sydney Australia

**Keywords:** caregivers, family, mutual‐help, SMART recovery, substance use disorders

## Abstract

**Introduction:**

Improving support for people affected by another's substance use disorder (affected family members [AFM]) is a priority, but implementation is challenging. This mixed‐methods evaluation assessed the perceived effectiveness of SMART Family and Friends training and applied the Consolidated Framework for Implementation Research (CFIR) to examine program implementation.

**Methods:**

*N* = 24 participants with professional or lived experience of supporting AFMs were recruited and trained to deliver groups. Training was evaluated using the Work Practice Questionnaire. Semi‐structured interviews (*n* = 19) captured experiences of training and group delivery. Transcripts were analysed using iterative categorisation, informed by CFIR domains.

**Results:**

Participants felt equipped to deliver groups (*M* = 20.79, SD = 3.09) and found the training useful (*M* = 28.40, SD = 2.74) and relevant (*M* = 27.16, SD = 2.85). The approach, applicability and structure of the intervention (intervention characteristics) promoted engagement. Workplace characteristics influenced time and resources available to support implementation, with adequate staffing paramount (inner setting). Participants identified a clear need for the program, but practical and attitudinal barriers complicated engagement (outer setting). Lived experience promoted facilitator engagement and also raised wellbeing and training considerations (individual characteristics). Challenges associated with delivery modality and experiences facilitating groups (implementation process) were identified.

**Discussion and Conclusions:**

SMART Family and Friends has potential for scaled implementation by peers and providers within voluntary positions and those employed within existing AOD and community service programs, although service limitations and barriers around help‐seeking may hinder delivery. Efforts to address training and infrastructure considerations, particularly among those with lived experience, are needed. Improved understanding of the support needs and preferences of AFMs is warranted.

## 1 Introduction

Internationally, it is estimated that more than 100 million people are impacted by the addictive behaviour of someone they care for [1]. Evidence suggests that affected family members (AFM) experience a broad range of adverse consequences, including reductions in their emotional, physical, social and financial wellbeing [2, 3, 4, 5]. Support for AFMs is limited, with lack of awareness, lack of services, hopelessness, previous negative experiences and stigma representing key barriers to help‐seeking [6, 7, 8]. Moreover, this population tends to delay help‐seeking [9] and thus effective, acceptable, and accessible support options for AFMs are required.

In the broader alcohol and other drug (AOD) treatment sector, emerging evidence highlights the importance of peer/lived experience support workers in treatment provision [10, 11]. Peer support interventions are associated with positive substance use outcomes, higher treatment engagement, increased self‐efficacy, increased confidence in recovery, and high treatment satisfaction [12, 13]. Preliminary evidence suggests that mutual‐help groups are effective for both primary AOD clients and their affected families [14, 15]. Mutual‐help groups are well placed to improve the sector's support for AFMs, given the demonstrated accessibility and stigma‐related barriers to help‐seeking.

SMART Family and Friends is a mutual‐help group explicitly focused on the wellbeing of AFMs. The program uses a strengths‐based and harm‐reduction approach informed by the SMART (self‐management and recovery training) four‐point program (understanding motivation, managing urges, problem solving, lifestyle balance). SMART Family and Friends draws from the evidence‐based stress–strain‐coping‐support model and accompanying 5‐step method [16]. The program comprises eight modules focused on promoting positive lifestyle changes, self‐care, assertive communication, and problem solving. Modules can be delivered flexibly based on the preferences and needs of participants and facilitators. For example, groups may be run as full‐day intensive sessions or as multiple 1–2‐h sessions delivered weekly.

Given the importance of improving the accessibility of dedicated support for AFMs [17] and preliminary evidence for the effectiveness of SMART Family and Friends [15], the current evaluation was designed to support broader implementation. Specifically, to assess the perceived effectiveness of SMART Family and Friends training for prospective facilitators and examine enablers and barriers to subsequent program implementation following completion of the training.

## 2 Methods

### 2.1 Study Design

The quantitative component of the study utilised an exploratory cross‐sectional study design, with data collected from participants at a single time‐point following participation in the facilitator training program. The qualitative component utilised semi‐structured interviews conducted after facilitators had delivered the SMART Family and Friends groups, or after approximately 2‐months following training (whichever came first). Findings are reported according to the Journal Article Reporting Standards for Qualitative Primary, Qualitative Meta‐Analytic, and Mixed Methods Research in Psychology [18].

### 2.2 Ethics Approval

Approval for the conduct of the study was provided by the University of Wollongong's Human Research Ethics Committee (UOW‐HREC; approval number: 2020/019).

### 2.3 SMART Family and Friends Training

Training was provided via Zoom in a series of single‐day (~8 h) workshops (5–8 participants per workshop) throughout January 2022. Sessions were led by the SMART Recovery Australia Senior National Program Manager and Trainer (located in Queensland) and observed by a researcher (AKB; located in New South Wales). Training was delivered to participants in volunteer roles and community‐based, not‐for‐profit providers of both specialised AOD support and broader health and wellbeing services. The training outlined the SMART Family and Friends program (rationale, theoretical underpinnings, principles, and therapeutic strategies) and group facilitation skills (via modelling, role‐playing, receiving feedback, and self‐reflection). Training was guided by the SMART Family and Friends facilitator manual. All participants were trained in the overarching SMART Recovery model prior to completing the Family and Friends facilitator training. The decision to facilitate the training online was compelled by COVID‐19 related mandates which limited movement and gathering. Providers were located in major cities (*n* = 10) and regional/remote (*n* = 13) regions of Western Australia, Victoria, New South Wales and Queensland.

### 2.4 Participants

**FIGURE 1:**
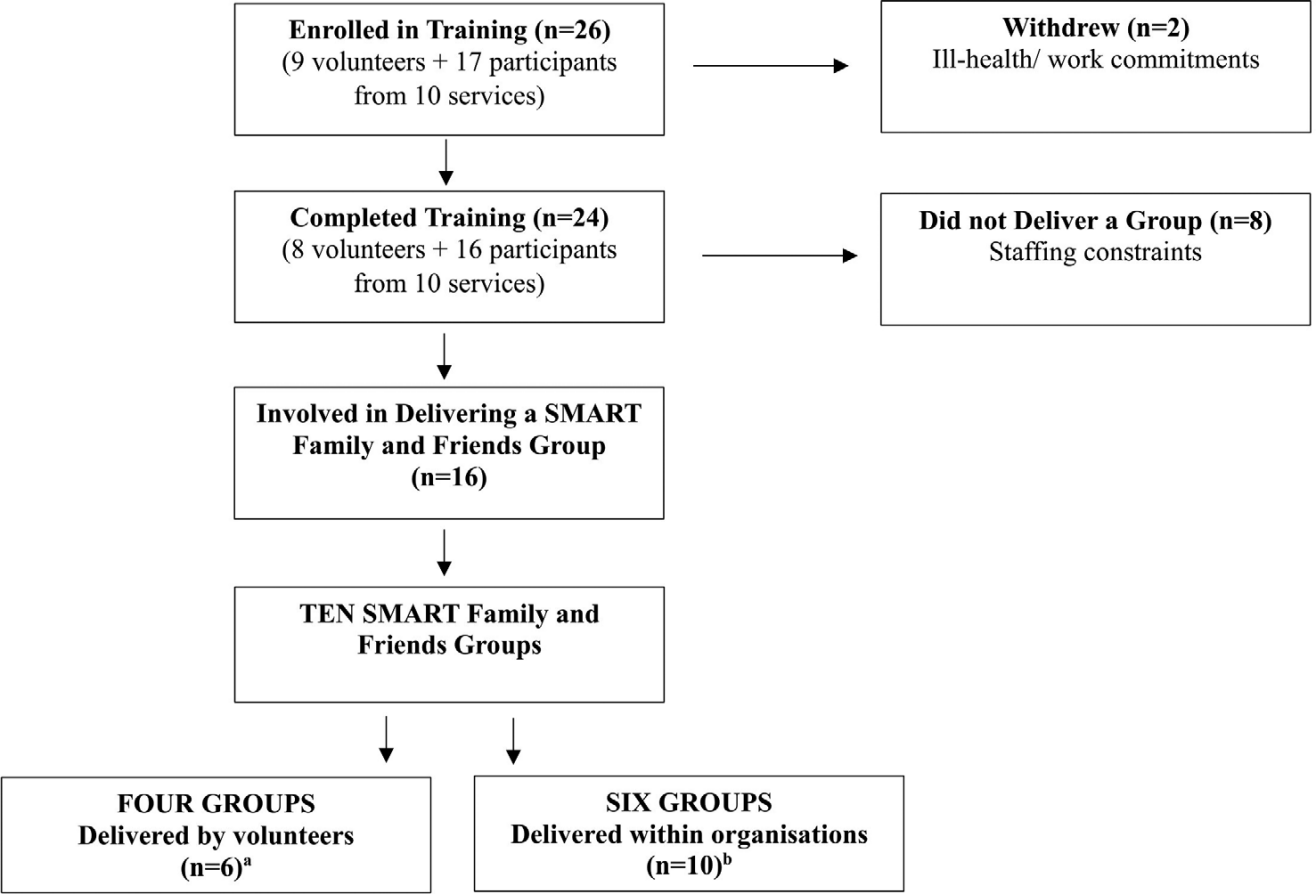
Study flow diagram. ^a^Two teamed up with a co‐facilitator; ^b^four teamed up with a co‐facilitator.

Participants (see Figure 1) were peers (lived experience as an AFM) and providers (professional experience supporting AFMs), aged 18 or older, who expressed interest in being trained to deliver SMART Family and Friends. SMART Recovery Australia advised current SMART Recovery facilitators about the study via email. The research team sent information via text/email to facilitators from a prior study [19] who provided consent to be contacted about future research. No participants in the study had previously attended training to deliver the SMART Family and Friends intervention. Training was provided free and all participants received a SMART Family and Friends Facilitator manual. An AUD$30 supermarket voucher was provided to interview participants to reimburse them for their time. All participants provided written or verbal informed consent.

### 2.5 Procedure and Data Collection

After completing facilitator training, participants' experiences were assessed using an anonymous online questionnaire. The questionnaire included items assessing participant demographics and selected subscales from the Work Practice Questionnaire (WPQ; described below). Between March and July 2022, qualitative interviews were conducted over the telephone by AKB with 19 participants, using a semi‐structured interview guide (see Appendix A) informed by the Consolidated Framework for Implementation Research (CFIR [20]). The CFIR comprises five domains (intervention characteristics, inner setting, outer setting, characteristics of individuals, process variables) and was used to conceptualise and classify the various complex factors that contribute to effective intervention implementation. Interview questions elicited participant perspectives on their motives for attending the facilitator training, experiences of the training, barriers and facilitators to intervention delivery, the helpfulness of various aspects of the intervention, and suggestions for improving the delivery and dissemination of the program.

### 2.6 Quantitative Measures/Variables

*Demographic characteristics* included age (25–34 years, 35–44 years, 45–54 years, 55–64 years), gender (female, male, non‐binary/gender fluid), postcode of residence (major city, regional/remote), country of birth (Australia, other) and highest level of education (Certificate III or IV, diploma, bachelor's degree, graduate certificate/diploma, master's degree). Items were selected from validated sources, including the National Minimum Data Set [21] and best practice recommendations for enquiring about gender [22].

*Professional characteristics* included professional background (social work, support work, counselling, case management/care coordination, peer work, external to AOD field), education/training (AOD, working with AFMs, peer work, mental health, trauma, disability), and personal experience with substance‐related or other addictive behaviours (personal lived experience, provider experience, both), and AFMs (personal lived experience, provider experience, both). Professional background and education items were derived from the WPQ. The item to capture lived experience was developed by the research team.

#### 2.6.1 WPQ

The WPQ is a validated [23] 109‐item questionnaire, comprising 19 subscales, used for the evaluation of training programs in the AOD sector [24]. Three subscales were administered: Role Adequacy, Perceived Training Outcomes, and Perceived Relevance of Training. All items were adjusted to reflect the objectives of the SMART Family and Friends training (e.g., “I have the necessary knowledge to help *affected family members*” instead of “I have the necessary knowledge to help people with *alcohol and other drug related issues*”). The Role Adequacy subscale is scored on a Likert scale, with responses ranging from 1 (*disagree*) to 4 (*agree*), and a total score ranging from 6 to 24. The Perceived Training Outcomes and Perceived Relevance of Training subscales are scored on a Likert scale, with responses ranging from 1 (*disagree*) to 5 (*agree*), and total scores ranging from 6 to 30. Item 5 on the Role Adequacy subscale, and Item 5 on the Perceived Relevance of Training are reverse coded.

### 2.7 Data Analysis

*Quantitative data analysis* was conducted using SPSS v28. Participant demographics were summarised using descriptive statistics. The WPQ subscales were scored according to published guidelines, treated as continuous variables and are reported as means and standard deviations.

#### 2.7.1 Qualitative Data Analysis

All interviews were audio recorded, labelled with a unique participant identifier and transcribed verbatim by a professional transcription service. Transcripts were checked for accuracy by AKB and imported into NVIVO 12 for organisation and analysis. Transcripts were analysed using iterative categorisation, a transparent, systematic approach to analysing qualitative data [25, 26] that can be applied to both deductively and inductively derived codes. Iterative categorisation is also compatible with other common qualitative approaches to analysis, including content analysis and thematic analysis. Deductive codes were developed by AKB based on the domains included in the CFIR and the broad study aims. These codes were then trialled using a subset of the interview transcripts, and a number of inductive, data‐driven codes were added. The resulting coding framework was then applied to all interview transcripts, with all textual data categorised into one or more codes and subsequently analysed to identify meaningful themes within each code. AKB conducted the initial coding and compiled the preliminary report. RMG conducted the second coding phase and identified the final themes and most pertinent quotes for publication, in consultation with the co‐authors and broader research team. The multi‐phased coding by two researchers constituted a verification process, in that concurrence was tested and resolved at each stage, collaboratively.

## 3 Results

### 3.1 Participant Characteristics

Participant characteristics are presented in Table 1. The average age of participants was 43.33 (SD = 11.84) years, more than half (14/24, 58.3%) identified as female and just over half (13/24, 54.2%) lived in regional or remote locations. Professional backgrounds included social work, support work, counselling, and lived experience. Most people (17/24, 70.83%) had attained a Bachelor's Degree or higher.

**TABLE 1:** Participant characteristics (*N* = 24).

	Total, *n* (%)
Age, years (*n* = 24)	
25–34	8 (33.3%)
35–44	6 (25%)
45–54	< 5
55–64	6 (25%)
Sex at birth (*n* = 24)	
Female	15
Male	9
Gender (*n* = 24)	
Female	14 (58.3%)
Male	8 (33.3%)
Non‐binary/gender fluid	< 5
Born in Australia (*n* = 24)	
Yes	20 (83.3%)
No	< 5
Location (*n* = 23)^a^	
Major city	10 (41.7%)
Regional or remote	13 (54.2%)
Professional background (*n* = 24)^b^	
Social worker	6 (25%)
Support worker (incl. psychosocial disability support)	5 (20.8%)
Counsellor	9 (37.5%)
Case manager or care co‐ordinator	6 (25%)
External to the AOD field	< 5
Peer worker/worker with lived experience	6 (25%)
Experience with affected family members (*n* = 24)	
Personal lived experience only	6 (25%)
Provider (paid or volunteer) experience only	< 5
Both	16 (66.6%)
Highest level of education and training (*n* = 24)	
Certificate III or IV	< 5
Diploma	< 5
Bachelor's degree	5 (20.8%)
Graduate certificate/diploma	6 (25%)
Master's degree	6 (25%)
Education or training with a significant component^b^	
Alcohol and other drugs	14 (58.3%)
Affected family and friends	9 (37.5%)
Peer work	< 5
Mental health	13 (54.2%)
Trauma	9 (37.5%)
Disability	5 (20.8%)

Abbreviation: AOD, alcohol and other drugs.

^a^ Missing data for *n* = 1 participant on this variable.

^b^ Participants allowed to select multiple categories.

### 3.2 Implementation

Of the 24 participants who completed training, 16 went on to deliver the SMART Family and Friends program in a volunteer capacity (*n* = 6) or through their organisation (*n* = 10; across six organisations). Groups were delivered using individual and co‐facilitation models, with a total of 10 conducted between March and September 2022.

### 3.3 Training

**FIGURE 2:**
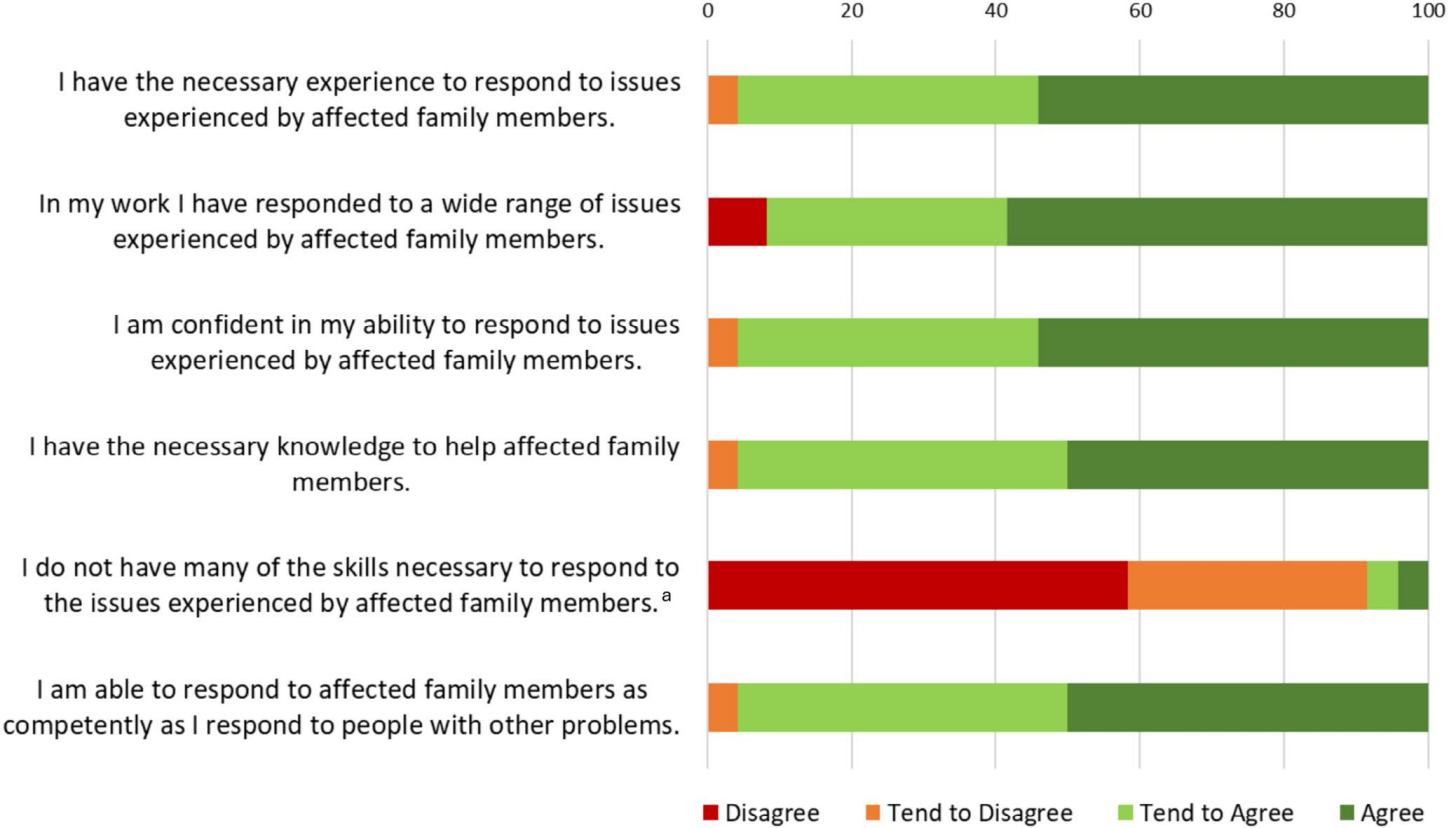
Participant responses to the ‘Role Adequacy’ items from the ‘Individual’ domain of the Work Practice Questionnaire. ^a^‘Disagree/tend to disagree’ on negatively worded items reflects a positive response.

**FIGURE 3:**
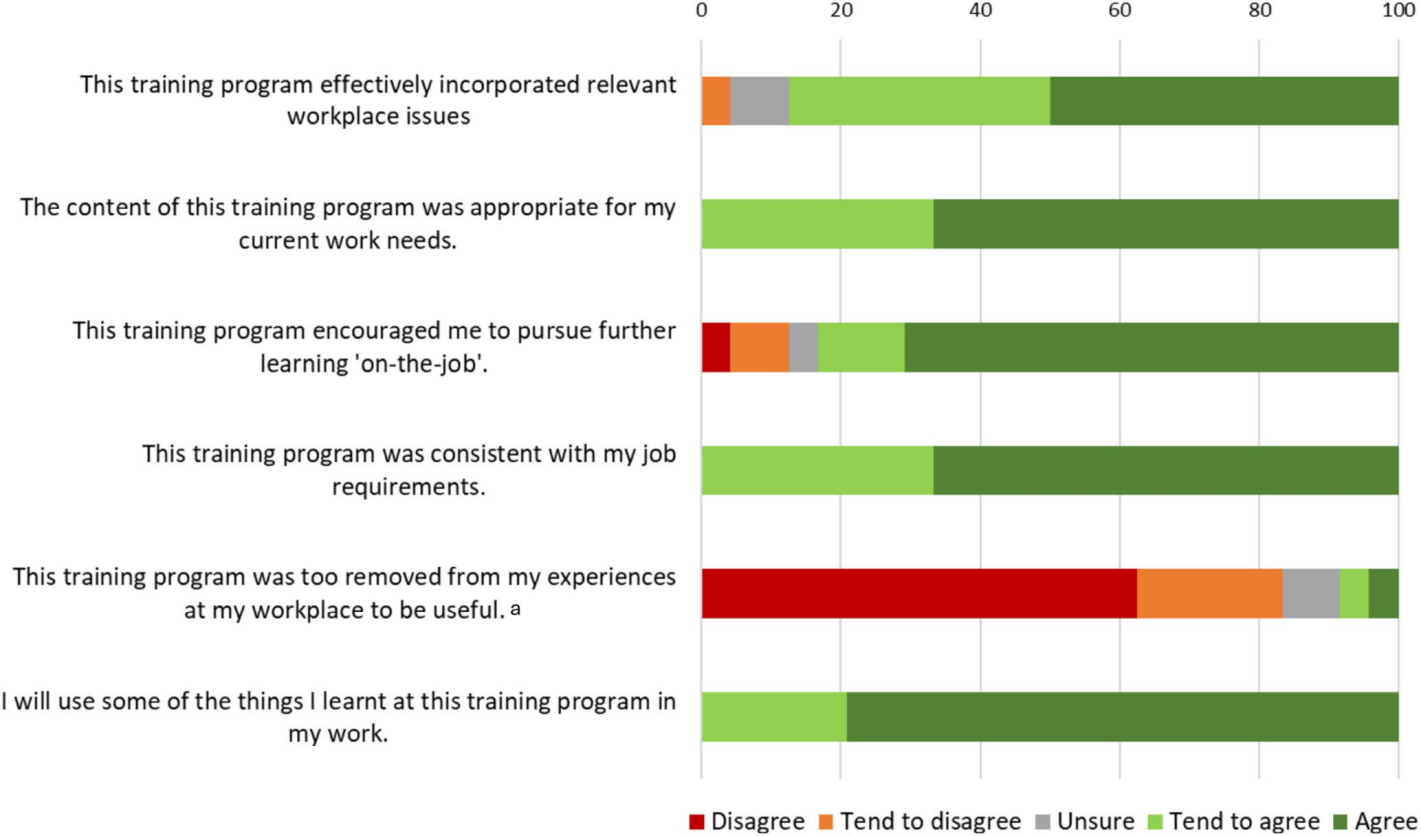
Participant responses to the ‘Perceived Relevance of Training’ items from the ‘Post‐training Section: Perceptions of Training’ domain of the Work Practice Questionnaire. ^a^‘Disagree/tend to disagree’ on negatively worded items reflects a positive response.

**FIGURE 4:**
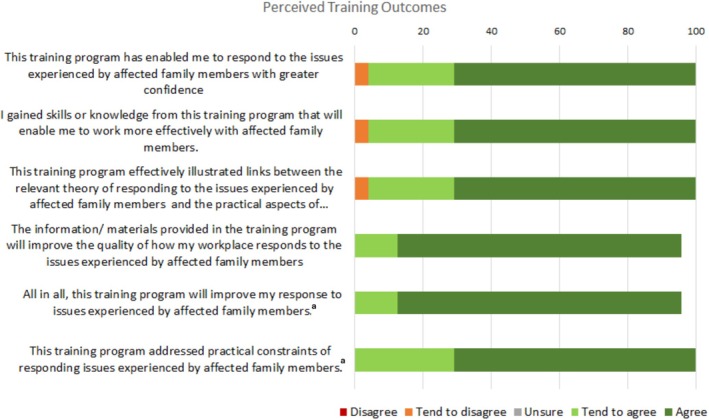
Participant responses to the ‘Perceived Training Outcomes’ items from the ‘Post‐training Section: Perceptions of Training’ domain of the Work Practice Questionnaire. ^a^Missing data for one participant.

Findings from the ‘Role Adequacy’, ‘Perceived Relevance of Training’ and ‘Perceived Training Outcomes’ subscales of the WPQ are presented in Figures 2, 3, 4. Mean score on the Role Adequacy subscale was 20.97 (SD = 3.09, range = 11–24), Perceived Training Outcomes subscale was 28.40 (SD = 2.74, range = 20–30), and Perceived Relevance of Training was 27.16 (SD = 2.85, range = 20–30). The proportion of positive responses (responses of ‘agree’ or ‘tend to agree’ on positively scored items, responses of ‘disagree’ or ‘tend to disagree’ on reverse‐scored items) ranged from 91.6% to 95.9% on the ‘Role Adequacy’ subscale, 83.3% to 100% on the ‘Perceived Relevance of Training’ subscale, and 95.8% to 100% on the ‘Perceived Training Outcomes’ subscale. All but two items were positively rated by more than 90% of respondents, with ‘this training program effectively incorporated relevant workplace issues’ and ‘this training program encouraged me to pursue further learning “on‐the‐job”’ being positively rated by 87.5% and 83.3% of respondents, respectively.

### 3.4 Intervention Characteristics

Intervention characteristics refer to the elements of the SMART Family and Friends program that influence its implementation, including the perceived adaptability, evidence base, intervention design and fitness for purpose [27]. Participants tended to have prior knowledge of the overarching program (SMART Recovery) and were drawn to the group due to their positive perception of the ethos, broad applicability, therapeutic benefits and structure of this approach. Participants also noted that the harm reduction orientation of SMART Recovery aligned with their professional and organisational values, which supported the program's integration into existing treatment programs.

It was the ethos of SMART Recovery. I really liked the messaging. I liked the core values of the programme […]. Really respectful language. No labels, naming, shaming. (#3)

The perceived relevance of the program to their broader work was also appealing, with many using the material in counselling for individuals and families.

It's probably one of my main booklets that I go to every now and again if I'm kind of stuck for an idea or what to do. […] And I think, yes, the content in there, it's really good. (#9)

Other factors which supported uptake by stakeholders included experiences of service gaps for affected family and friends, and the potential for SMART Family and Friends to address this need for targeted support and psychosocial education. Participants also shared their observations about positive client outcomes, such as adopting self‐care techniques or setting improved boundaries. Facilitators were pleased to observe AFMs adopting a greater capacity to choose how they respond to their loved one, with perceived positive outcomes for AFMs a central consideration in ongoing implementation.

And a lot of the participants came, and they were really deflated in the beginning. Especially when you bounce around ideas, like setting up boundaries and things like that, we've tried all of that, that doesn't work. We've tried everything. This has been a 17‐year issue. So, to see the transition from that to the end, if we knew this stuff 10 years ago, we would have avoided a whole lot of stuff. (#14)

Having a structured manual was considered particularly beneficial for newer group workers, as was experiential elements of training that allowed participants to practice what they were learning. The manual helped to bolster confidence by summarising the content, timing and structure of each session, while experiential activities afforded the opportunity to practice key skills in a safe space and receive immediate feedback.

I really liked that it's structured. Every week has one particular thing that we talk about. And also, the training in general was good, because we were also encouraged to do the practical thing within the training, be the facilitators. (#6)

### 3.5 Inner Setting

Within the CFIR, Inner Setting characteristics refer to the characteristics of the setting in which the intervention is implemented and how these affect program implementation (27). A lack of paid time and a lack of resources or support to establish a group were key comments, and managing these challenges was described as a “logistical nightmare” (#12). Participants described efforts to be responsive to clients with differing needs and availability; however, this resulted in difficulty determining the schedule and structure of the group.

Probably just the logistics of, when's the best time to run it, to cater for everyone. And, do you do two full days, or do you do one each week? (#21)

Having access to adequate rooms to host meetings, combined with the administrative burden of diarising a meeting, was particularly fraught. Although participants recognised a need for the SMART Family and Friends program, already stretched building capacity posed a barrier in some services. In these contexts, it is likely that interventions to support AFMs are competing for limited space and time with pre‐existing groups in which AFMs are not the target population.

It's the availability of rooms. […] Just the space for it, yes, because multiple times I've tried to book that one. We don't have very many big rooms. There's a couple, but they're always booked out, because there's so many groups running. (#6)

Likewise, where participants were working within well‐resourced settings, the practical components of group service delivery were deemed to be more feasible, further pointing to physical space and resources as both a key barrier and facilitator to sustainable program implementation.

I suppose it depends where people are going to run it because as I said, we're lucky here because we have a really good space. (#10)

High staff turnover and short‐term funding cycles within allied health services also increased the barriers to implementation. Where support for AFMs is not a core element of a service, it may be among the services to be discontinued when staffing and caseload issues arise, further hindering support for a population with limited access to resources.

We've had high staff turnover, which has led to increased caseloads and us being unable to offer maybe some of those supplementary services that we previously had been able to do. (#12)

Study participants had varied levels of management support, and executive endorsement of the program was not consistently matched by active participation. A number of participants expressed a dissonance between their interest in running the group and the prohibitively high administrative burden of implementation. This was thought to compound professional challenges relating to time poverty and professional performance anxiety.

The biggest challenge for us at the moment is we're very short‐staffed, so our team are all carrying quite a large caseload, so for us at this point in time, the thing would be time pull, which has made it a bit challenging. Under different circumstances, we might have carried a lesser caseload, would've had time to just devote to organising things. (#5)

Given the challenges that participants faced in staffing these groups, online facilitation was proposed to overcome trained‐staff shortages. However, the motivation and capacity to facilitate online meetings was varied, with online implementation described as both an opportunity to improve access and equity and as a potential barrier to active program participation (described below, see ‘Section 3.8’).

### 3.6 Outer Setting

This domain was used to collate interview excerpts which spoke to the external, societal facilitators and barriers for program implementation. As highlighted in the first section, participants were motivated to trial the program within their service due to their awareness of unmet support needs within this cohort.

Well, we were aware of the normal SMART that's running in the community. And as practitioners, we've obviously identified that there's such a huge need for the people whose loved ones are using, they're obviously highly impacted as well. (#6)

Social isolation, due to care giving responsibilities, and stigma, associated with “addiction,” were perceived as key challenges for affected family and friends, and as such a mutual‐help group was considered a good fit. However, despite the willingness of facilitators to run the program, attracting sufficient participants to run the group was a common challenge. Central to this, the secondary stigma factor was described as a key barrier to help‐seeking for affected family and friends.

To get our group up and running there's still a massive stigma barrier. […] The general population still looks at drugs and alcohol as a massive problem. It's demonised and villainised and it's, everyone's a junkie. (#9)

Participants also described how AFMs might not need support, or if they did, might experience difficulties acknowledging that need. Participants talked about the resilience of AFMs and how they may already have skills, strategies and support networks in place, thereby reducing the likelihood that they would engage with the SMART Family and Friends program. Thus, participants acknowledged that while there is a definite need for increased support for AFMs in the community, it is important that the individual context and needs of AFMs are considered as the need for structured support is not a universal experience.

But there's a very real potential that people have their own way of dealing with someone who's got substance issues. […] So they may have developed their own coping strategies that are just as beneficial. (#5)

Attitudinal barriers (e.g., needing to remain ‘strong’; not wanting to ‘air dirty laundry’) were also raised as considerations affecting the engagement of AFMs, with limited uptake among AFMs having the potential to adversely affect the program's ongoing delivery. Distinguishing between a genuine lack of need for support and stigma‐related barriers to help‐seeking is a key challenge for service providers, and highlights the importance of individualised referral and support approaches.

Resource deficiencies were also identified as barriers, such as a lack of stable housing or work. This challenged program attendance, and study participants were required to account for these issues when establishing the group.

Not only the time commitment but if they're having to catch public transport, then there's a cost commitment there. If they're […] got job seeker responsibilities that they need to fulfil that may take time. And then, if they themselves are homeless, there's trying to find a roof over their head […] And whilst they might want to be able to support their loved one better, it's probably just not at the top of the list to be in every week for eight weeks or for the other option of two days. (#20)

### 3.7 Individual Characteristics

This domain captures the characteristics of the individuals involved in the implementation of an intervention which may impact the implementation process [27]. Thus, having described their perceptions and experiences of the SMART Family and Friends Group, participants were invited to share what it had been like for *them* to undertake the training and implementation of the program. Our sample included professionals who had personal experiences of problematic AOD use, and/or informal caregiving, and often cited this as a reason for seeking training in the SMART Family and Friends program. However, having personal experience was also thought to increase burnout risks and participants questioned their ongoing objectivity.

Obviously, having a family member that also struggles with addiction, you still have the frustration of everything, so I found that it might be a little bit much for me. (#7)

Study participants, whether they indicated that they had previous experience or not, repeatedly described transferring what they learnt in SMART groups to their professional lives, as well as their personal lives. This has the potential to mediate the risks of burn out, in that participants reported an increased understanding of their own support needs through facilitating the groups.

And also support for family and friends, I think I found it was helpful for the whole family. I think it made me a lot more positive about the situation and in terms of handling siblings and whatever, that was really useful. (#17)

### 3.8 Implementation Process

So far, we have presented interview excerpts to describe the intervention model, the host service, the effect of societal factors, and the facilitator. In this section, the focus shifts to the process of implementation and how these factors influenced the provision and effectiveness of the program (referred to as the “Implementation Process” domain within the CFIR [27]). There were repeated accounts of client challenges in service access due to a lack of transport and/or childcare, particularly for mothers with young children. This complicated implementation efforts, as successful and equitable implementation required that additional services and supports be introduced in conjunction with SMART Family and Friends groups.

If the parents were coming to the programme, presumably they were going to have to find someone to look after their children. And so, part of what we had to take on to run the programme was to be able to provide [childcare]. (#10)

In addition to addressing resourcing issues (described above, see ‘Section 3.5’), online groups were suggested as a means of overcoming these access issues.

I think you'd have better attendance on Zoom because people can be at home and say, oh, I've got my meeting in five minutes I'll just go and jump on it. And everyone seems to be quite comfortable too, whereas if they had to drive half an hour to somewhere to a meeting you may not get that. (#17)

Participant appetite for and experience with online mechanisms, however, was varied, with concerns about active engagement and participation in online meetings cited by participants. Participants expressed concerns about whether group members would feel comfortable to actively contribute in online sessions, and suggested that distractions while participating from home may also hinder the effectiveness of their participation. Facilitators' competence and confidence using online platforms to deliver the program was also raised as a potential barrier to successful implementation. As such, in‐house training and support to use digital platforms was recommended to improve the professionals' confidence in delivering the program online without requiring large investment of resources.

I had never really used Zoom so I actually got my manager at the time, we were having some downtime at work and I asked him whether or not he'd ever used Zoom. And he had a play with me in terms of that's how you do this and this button is for this. It was not even 10 minutes kind of thing, but you need confidence to be able to do that. (#7)

Professional confidence in managing the later modules of the program, which explore personal and family safety and domestic violence, was less reliable, due largely to the potentially ‘triggering’ nature of this content. While domestic and family violence information was seen as appropriate for this client cohort, participants were less confident about facilitating this material and, as such, additional training for this topic was recommended. Indeed, this was a topic where co‐facilitation with an experienced colleague was described as paramount.

We had a good discussion around the types of violence, what's considered to be family and domestic abuse. A lot of which these people have suffered, but they didn't know it was domestic abuse. So, that was, you know. And [co‐facilitator] handled it with great sensitivity, even before we went into the content. Saying, this is quite heavy. This is quite triggering. Please let us know at any time if this gets a bit much, and we'll do something else. (#13)

Training around communicating about domestic and family violence, then, has the potential to improve confidence and mastery among facilitators, who may in turn impart additional awareness to AFMs. Given the proportion of peer‐workers within this cohort, this has the potential to help them know what they too are living with, and how best to treat any associated distress. However, there remains the potential that facilitators lacking expertise or confidence in communicating about these issues may contribute to unintentional distress among AFMs, further highlighting the importance of effective training and supervision for novice facilitators.

## 4 Discussion

This study assessed the perceived effectiveness of SMART Family and Friends training among prospective group facilitators and examined enablers and barriers to program implementation. The training and support were perceived to be adequate, with facilitators describing high levels of readiness, knowledge and confidence to deliver SMART Family and Friends. Training considerations around online delivery and sensitive topics (e.g., family violence) were raised, particularly for volunteers new to group facilitation. Although our findings support the implementation potential of SMART Family & Friends by peers and providers within voluntary positions and those employed within existing AOD and community service programs, program uptake was impacted by service capacity and practical and attitudinal barriers to help‐seeking among AFMs.

We identified several characteristics of SMART Family and Friends that supported implementation. Aligned with published findings [28] facilitators described a clear need for improved support options for AFMs. SMART Family and Friends was therefore viewed as an opportunity for addressing a widespread, long‐standing gap in service provision. Facilitators also appreciated the structured, non‐judgemental format of the program, consistent with previous research identifying the importance of these elements for engaging AFMs [29, 30]. Finally, the focus on experiential learning during training and explicit guidance provided in the facilitator manual were beneficial. These findings align with previous research demonstrating the importance of experiential learning for training healthcare providers [31] and peers [32], and the benefits of combining active (e.g., role‐play) and passive (e.g., treatment manuals) training methods for skills development [33]. Given that study participants also expressed some uncertainty regarding online facilitation, group process and navigating sensitive topics, integrating experiential learning into ongoing supervision and coaching would likely help to bolster self‐efficacy, particularly among those new to group facilitation. This is especially important given recent findings for the importance of the facilitator to the in‐group experience of AFMs [34].

Consistent with published findings [35], ‘inner‐setting’ service‐related factors such as managerial support and adequate resourcing/facilities were integral to program implementation. Even in the presence of managerial support and a clear need for the service, implementation was complicated by resourcing and infrastructure considerations. Healthcare initiatives are frequently hindered by unpredictable and constrained funding [36]. Moreover, this study was completed during the COVID‐19 pandemic, which likely exacerbated resourcing concerns. Further research is needed to quantify the cost of delivering the SMART Family and Friends program, as well as the broader resourcing required to support implementation and dissemination. Comprehensive guidance is available to support this work [37].

Outer‐setting factors, including stigma and cultural attitudes around remaining ‘strong’ in the face of adversity, were identified as key barriers to group implementation. Stigma is a pervasive concern for individuals experiencing SUDs [38] and extends to AFMs [7, 39]. Self‐stigma (internalised negative attitudes and shame resulting from exposure to stigmatising beliefs and behaviours) is also a concern for people who use drugs [40] and likely impacts AFMs similarly. Consistent with findings of a recent systematic review [41], our results suggest that self‐stigma may compound the effects of public stigma in reducing help‐seeking among AFMs. This is despite the potential for interventions involving peers to reduce stigma [29]. Stigma‐related barriers to AFM engagement were exacerbated by pragmatic considerations such as childcare, employment, and service accessibility. To better support engagement and retention, efforts to identify the help‐seeking needs, preferences and experiences of AFMs are warranted.

While AFMs may experience wide‐ranging adverse outcomes as a result of their loved one's substance use [41], it is important to recognise the strengths and resilience held by families to manage these challenges. Participants in the study acknowledged the creative ways that AFMs access support from their communities and the possibility that not all AFMs need formalised support. Previous research has similarly identified the strengths of AFMs and the ways that these strengths may be applied to caring for both themselves and their loved ones [2, 6]. Conversely, AFMs may need support but be unaware of the options available to them [7, 8]. Consistent with the principles of person‐centred care [42], our findings highlight the need for an individualised approach to supporting AFMs. Specifically, a focus on improving awareness of support options is needed for those with limited experience navigating the treatment system; individual barriers to help‐seeking should be addressed; intervention delivery should be tailored to overcome access barriers and ensure equity of access; and the existing strengths and resources of individual AFMs must be acknowledged. Support options such as SMART Family and Friends may not be appropriate or necessary for all AFMs, and the individual needs and context of each client must be considered when developing an approach to support.

Online delivery was discussed as a potential strategy to overcome identified implementation barriers, however, concerns about group engagement and commitment in an online format were also raised. Preliminary findings support the feasibility of online delivery of mutual‐help groups for AFMs [29, 34]. However, evidence from people experiencing problematic alcohol use demonstrates that although attendance frequency may increase, engagement may be lower when mutual help groups are delivered online [43]. This may reflect differences in the clients attracted to online compared to face‐to‐face services [19], or by features of the online experience itself (e.g., technology glitches, confidence with technology, reduced non‐verbal communication, access to private space to attend sessions; [44]). Little is known about the relationship between group modality and outcomes for AFMs, and identifying the factors that promote or impair engagement for this population represents an important challenge for future research. It is also essential that use of technology to deliver treatment and support does not disproportionately disadvantage those already at risk of poor outcomes (e.g., people in rural locations, older people).

While a growing body of research supports the use of mutual‐help and peer‐led interventions in the AOD sector [10, 45, 46], surprisingly, there is limited evidence for the personal impact and specific training needs required to support this dual‐role, particularly among AFMs [32]. Participants in the current study acknowledged the challenges associated with being a lived‐experience facilitator, particularly around the delivery of potentially triggering topics. This mirrors the experience of peer‐support workers in previous research [47]. Engagement in peer work may positively influence the lives of lived‐experience workers [48, 49], however, the benefits must be balanced against the possibility for distress and burnout. In the USA, the Substance Abuse and Mental Health Services Administration (SAHMSA) has developed core competencies in the provision of peer‐delivered services, including ongoing supervision and reflection on personal reactions/feelings elicited by peer‐support work [50]. To effectively manage the dual role of lived experience and supporting others, access to ongoing supervision and support is important.

Our findings need to be considered in light of several limitations. First, to minimise burden, administration of the WPQ was restricted to one sub‐scale of the ‘Individual’ domain and both subscales of the ‘post‐training’ scales. Although it is appropriate to tailor WPQ administration to the most relevant scales, doing so limits our objective assessment of factors that influence the transfer of training to workplace settings. Psychometrics for this tool are also limited, and benchmarks have not been published. Second, the vast majority of participants had extensive education, training and experience within addiction settings. Further evidence is needed to understand the needs and experience of those who are newer to group facilitation. Finally, our findings are derived from participant self‐report. Future research would benefit from objective evaluation of facilitator knowledge, adherence and competence (e.g., via objective rating of live or recorded role‐plays and session delivery).

## 5 Conclusions

Accessible and engaging support options for AFMs are needed. Our findings provide preliminary support for the implementation potential and scalability of the SMART Family and Friends mutual‐help program. To optimise delivery, dissemination and uptake, efforts to clarify and address training and infrastructure considerations, particularly among those with lived experience, are needed. Given the diverse experiences, contexts, and needs of AFMs, additional research exploring SMART Family and Friends implementation in diverse settings and longitudinal examinations of the long‐term sustainability and outcomes of the program are needed. Improved understanding of the support needs and preferences of AFMs is also warranted.

## Author Contributions

Authorship follows ICMJE recommendations (ICMJE, 2024). All authors made substantial contributions to the conception or design; or the acquisition, analysis or interpretation of the data. **Alison K. Beck:** conceptualisation, data curation, formal analysis, funding acquisition, investigation, methodology, project administration, writing – original draft, writing – review and editing. **Rebecca M. Gray:** data curation, formal analysis, writing – original draft, writing – review and editing. **Megan Wells:** writing – original draft, writing – review and editing. **Frank P. Deane**, **Briony Larance**, **Leanne Hides**, **Victoria Manning**, **Amanda L. Baker**, **Anthony Shakeshaft**, **Elizabeth Dale**, **Angela Argent:** conceptualisation, methodology, funding acquisition, writing – review and editing. **Peter J. Kelly:** conceptualisation, methodology, funding acquisition, supervision, writing – review and editing. All authors offered critical revisions to the manuscript for important intellectual content, have approved the final version of this manuscript and agree to be accountable for all aspects of the work in ensuring that questions related to the accuracy or integrity of any part of the work are appropriately investigated and resolved.

## Funding

This research was delivered through the Alcohol and Drug Foundation's Information Services and Support Program for Family and Friends (ISS R03), funded by the Australia Government to improve drug information and services for families.

## Ethics Statement

The study was approved by the University of Wollongong Human Research Ethics Committee (2020/019). All participants provided informed written or verbal consent prior to enrolment in the study.

## Conflicts of Interest

This research was delivered through the Alcohol and Drug Foundation's Information Services and Support Program for Family and Friends (ISS R03), funded by the Australia Government to improve drug information and services for families. Each author certifies that their contribution to this work meets the standards of the *International Committee of Medical Journal* Editors.

## Data Availability

The data is not available as participants of the study did not give written consent for their data to be shared publicly.
